# Circulating and Platelet MicroRNAs in Cardiovascular Risk Assessment and Antiplatelet Therapy Monitoring

**DOI:** 10.3390/jcm11071763

**Published:** 2022-03-22

**Authors:** Grzegorz Procyk, Dominika Klimczak-Tomaniak, Grażyna Sygitowicz, Mariusz Tomaniak

**Affiliations:** 1 First Department of Cardiology, Medical University of Warsaw, Banacha 1A, 02-097 Warszawa, Poland; grzegorzprocyk@gmail.com (G.P.); mariusz.tomaniak@wum.edu.pl (M.T.); 2 Department of Cardiology, Hypertension and Internal Medicine, Medical University of Warsaw, Kondratowicza 8, 03-258 Warszawa, Poland; 3 Department of Immunology, Transplantation and Internal Medicine, Medical University of Warsaw, Nowogrodzka 59, 02-006 Warszawa, Poland; 4 Department of Clinical Chemistry and Laboratory Diagnostics, Medical University of Warsaw, Banacha 1A, 02-097 Warszawa, Poland; gsygitowicz@poczta.onet.pl

**Keywords:** miRNA, antiplatelet therapy, biomarkers, platelets

## Abstract

Micro-ribonucleic acids (microRNAs) are small molecules that take part in the regulation of gene expression. Their function has been extensively investigated in cardiovascular diseases (CVD). Most recently, miRNA expression levels have been suggested as potential biomarkers of platelet reactivity or response to antiplatelet therapy and tools for risk stratification for recurrence of ischemic evens. Among these, miR-126 and miR-223 have been found to be of particular interest. Despite numerous studies aimed at understanding the prognostic value of miRNA levels, no final conclusions have been drawn thus far regarding their utility in clinical practice. The aim of this review is to critically appraise the evidence on the association between miRNA expression, cardiovascular risk and on-treatment platelet reactivity as well as provide insights on future developments in the field.

## 1. Introduction

Cardiovascular diseases (CVD) remain the most common cause of death worldwide, accounting for 4.1 million deaths per year among European Society of Cardiology (ESC) member countries [1]. Despite considerable advancements in CVD risk stratification and management over the past decades, there remains an urgent need for improved and early identification of subjects at high-risk of cardiovascular events [2].

The diagnosis of acute coronary syndrome (ACS), comprising ST-elevation myocardial infarction (STEMI), non-ST-elevation myocardial infarction (NSTEMI) and unstable angina (UA) [3], requires fast, sensitive and specific diagnostic, as well as prognostic tools. High-sensitivity cardiac troponins (hs-cTn) have emerged to be of a great value with respect to diagnosis of myocardial infarction (MI) and hence directing towards an invasive management strategy. Still, there are no widely accepted biomarkers available to guide optimal selection of dual antiplatelet therapy (DAPT) [4].

DAPT consisting of the combination of aspirin and a P2Y_12_ receptor antagonists—such as clopidogrel, prasugrel or ticagrelor—represents the cornerstone of ACS management. Nevertheless, some ACS patients exhibit impaired or exaggerated response to antiplatelet agents. The presence of high platelet reactivity (HPR), as assessed by platelet function testing, is associated with an increased risk of recurrent ischemic events in the setting of patients undergoing percutaneous coronary intervention (PCI) [5]. On the other hand, “hyper-responsive” individuals might be at increased risk of bleeding complications, in particular while on more potent prasugrel or ticagrelor therapy. Nevertheless, the assessment of platelet aggregation in vitro may not directly reflect in vivo processes [6,7,8]. Genetic testing has also been proposed as a strategy to help predict antiplatelet drug response [6,9,10]; however, genetic variants only contribute modestly to antiplatelet drug response and other factors may have a role [9,11]. Since interventional studies based on platelet function assays were largely negative to date, new biomarkers that capture other facets of platelet function regulation may better assess the thrombotic risk [12]. This can aid with a more individualised approach towards the selection of antiplatelet therapy, which may in turn improve outcomes [13]. To this extent, microRNAs (miRNAs) have emerged as potential biomarkers to be considered for the assessment and monitoring of the antiplatelet drug response [14,15]. The aim of this review is to critically appraise the evidence on the association between miRNA expression, cardiovascular risk and on-treatment platelet reactivity, as well as provide insights on future developments in the field.

## 2. MicroRNA: General Characteristics

**Figure 1:**
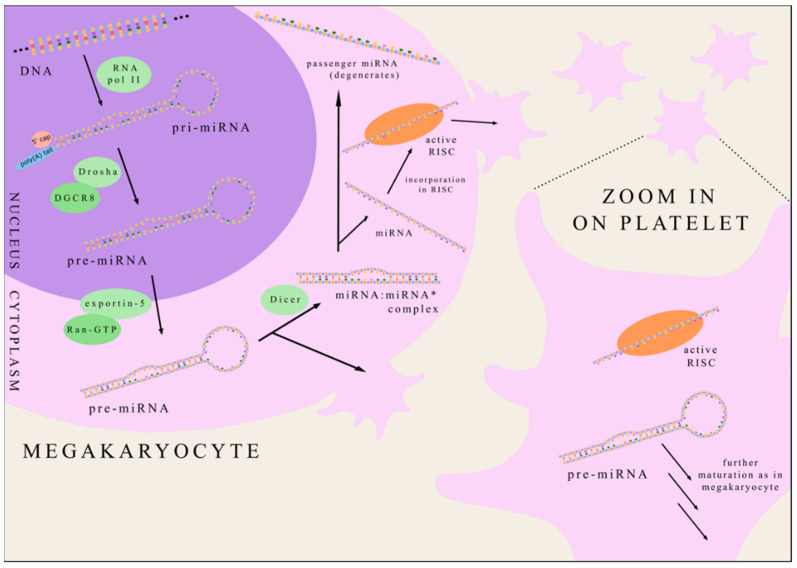
The biogenesis of platelet miRNA. miRNA—microRNA; RNA pol II—RNA polymerase II; pri-miRNA—primary-miRNA; pre-miRNA—precursor-miRNA RISC—RNA-induced silencing complex; DGCR8—DiGeorge syndrome critical region 8 protein.

MicroRNA is a small non-coding RNA consisting of approximately 22 nucleotides, first discovered in 1993 in nematode species *Caenorhabditis elegans* [16]. At present, almost 2000 various human miRNAs have been identified according to miRbase [17]. MicroRNAs are ordinarily transcribed in the nucleus by RNA polymerase II generating primary-miRNA (pri-miRNA) [18]. Pri-miRNA contains a 70 nucleotide-long hairpin structure recognised and processed by the enzyme Drosha (associated with a DGCR8 protein) forming precursor-miRNA (pre-miRNA) [19]. Pre-miRNA is then transported from nucleus to cytoplasm by eportin-5 in collaboration with Ran-GTP [20]. The cytoplasmatic endoribonuclease Dicer cleaves the pre-miRNA hairpin creating miRNA:miRNA* complex [21]. The following unwinding of this duplex creates two single-stranded miRNAs. Most commonly, one strand (termed “guide strand”) is incorporated into the RNA-induced silencing complex (RISC) and plays a role in modifying gene expression while the other strand degenerates (it is called “passenger miRNA” and marked as miRNA*). However, in some cases, passenger miRNA can also become functional in regulating gene expression [22]. Platelets inherit from megakaryocytes both mature miRNA, as well as pre-miRNA, which is further processed by Dicer and other enzymes such as in megakaryocytes [23] (Figure 1).

The key function of miRNA is the regulation of gene expression. MiRNA binds to target mRNA, thus downregulating translation [22]. Most commonly only about eight nucleotides are complementary triggering mRNA silencing. However, sometimes, nearly the entire miRNA is pair-matched leading to mRNA degradation. MiRNAs target about 60% of human genes [24]. Various miRNAs can be found not only inside the cells but also in the extracellular fluid constituting circulating miRNAs [25].

## 3. MiRNA in Coronary Heart Disease

Platelets are non-nucleated fragments of cytoplasm derived from the megakaryocytes [26]. Not only do they inherit mRNA and translation machinery, they are also regulators of gene expression [27]. In 2009, platelets were proven to harbour a plentiful and varied array of miRNAs [28]. Since that the discovery plenty of research has been undertaken focusing especially on variations in miRNA levels in different conditions [29,30,31]. Investigations concerned both platelet and circulating miRNAs. Importantly, miRNAs present in plasma exist in different forms: as protein-miRNA complexes or loaded into microvesicles or lipoprotein complexes [32].

**Figure 2:**
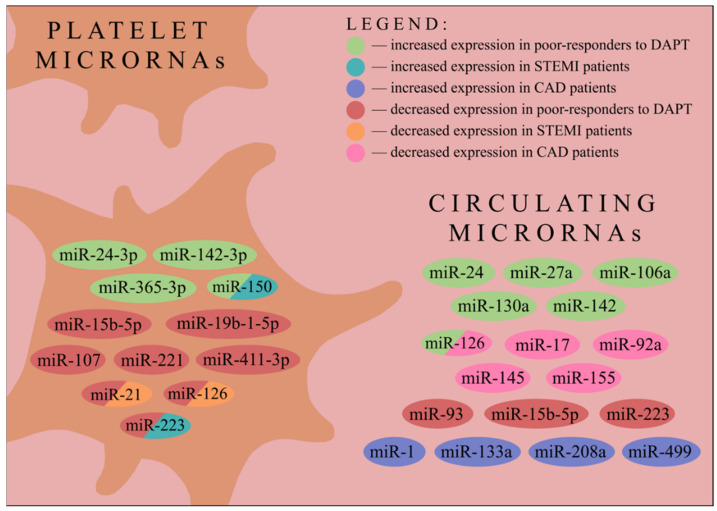
Changes in platelet and circulating miRNAs expressions in patients defined as poor-responders to dual antiplatelet therapy and in patients suffering from STEMI or CAD. miR/miRNA—microRNA; STEMI—ST-elevation myocardial infarction; CAD—coronary artery disease; DAPT—dual antiplatelet therapy.

Circulating miRNAs, as well as platelet miRNAs, have been extensively studied, with several correlations reported to date. Patients suffering from coronary artery disease (CAD) have reduced levels of circulating miR-126, miR-17, miR-92a, miR-155 and miR-145 in comparison to healthy volunteers, whereas miR-133a and miR-208a levels have shown to be elevated [33]. Another study demonstrated decreased expression of miR-147 (obtained from peripheral blood mononuclear cells) and increased levels of miR-135a in CAD patients compared to controls [34]. Platelet miR-21 and miR-126 were downregulated, whilst miR-150 and miR-223 were upregulated in ST-segment elevation myocardial infarction (STEMI) patients compared to the healthy control. Nevertheless, only miR-126—recently validated as platelet reactivity regulator—correlated with plasma cTnI [35,36]. Circulating levels of miR-1, miR-133a/b, miR-208a and miR-499 were suggested to be potentially useful in acute myocardial infarction diagnosis [37] (Figure 2).

MiR-378 and let-7b were significantly upregulated in mobilized CD34^+^ progenitor cells isolated from patients with STEMI compared to stable CAD patients and healthy controls. Moreover, miR-378 was found to be a crucial regulator of the proangiogenic activity of CD34^+^ progenitor cells [38]. A lot of other research assessed miRNAs as biomarkers of different CVD, expanding our knowledge about these small molecules [39].

## 4. Acetylsalicylic Acid and Clopidogrel Impact on Platelet miRNA Expression

Aspirin, in combination with a P2Y_12_ inhibitor, constitutes an essential treatment in patients with CAD [40]. Clopidogrel is the most commonly utilized P2Y_12_ inhibitor. However, some patients present with inadequate response to clopidogrel, which might be associated with an increased risk of cardiovascular events. It is estimated that about 4% to 30% of patients do not respond to DAPT adequately [41]. More potent P2Y_12_ inhibitors such as prasugrel or ticagrelor are advocated, however, at the expense of the higher bleeding risk [42]. Hence, meticulous assessment of the risk and potential benefits at the time of DAPT treatment initiation is crucial. Importantly, recent European Society of Cardiology guidelines suggested that the evaluation of the response to antiplatelet regimens might be instrumental in guiding treatment decisions (i.e., P2Y_12_ inhibitor de-escalation in the late phase after myocardial infarction) [40].

For these reasons, novel biomarkers evaluating the probability of inadequate response to antiplatelet therapy are continuously required. In this context, the potential utility of platelet miRNA for identification of patients with anticipated high on-treatment platelet reactivity is gaining an increased interest. Aspirin treatment in diabetes mellitus type 2 (DM2) patients resulted in a decreased level of circulating miR-126, most likely due to an inhibitory effect on platelet activation and miRNA-containing microvesicle release [43]. Healthy volunteers treated with aspirin or aspirin in combination with prasugrel presented decreased levels of miR-126, miR-150, miR-191 and miR-223 assessed by quantitative real-time polymerase chain reaction (qPCR) in platelet-poor plasma (PPP) [44]. Noteworthily, the circulating levels of the three aforementioned miRNAs, i.e., miR-126, miR-150 and miR-223, appeared to decrease whereas the level of miR-96 appeared to increase after switching from clopidogrel to prasugrel in DAPT [45]. All these findings indicate that different therapies, including DAPT, may influence miRNA concentration in the plasma, both via impact on their generation by megakaryocytes and the miRNA-containing macrovesicles release. Consequently, it may impede drawing firm conclusions about correlation between miRNA expression and platelet reactivity in further discussed research as, most often, investigated patients were under DAPT.

## 5. MiRNAs Expression and Platelet Reactivity

Resistance to clopidogrel (and other P2Y_12_ inhibitors) can be assessed by measuring platelet reactivity while on treatment. High on-treatment platelet reactivity is identified when platelet reactivity remains elevated despite applied therapy [46]. In particular, substantial interindividual variability in response to clopidogrel has been reported. However, the evaluation of platelet response to DAPT is challenged by the fact that it requires therapy to be continued for a given time period before blood biomarker analysis. In addition, the pharmacokinetics related to the time between drug administration and blood sampling need to be considered. Several studies to date aimed to identify an improved biomarker for cardiovascular risk stratification and assessment of platelet reactivity, among them miRNAs are currently becoming the centre of scientific attention [47]. Indeed, several correlations between miRNA concentrations and platelet reactivity have been found [36,48,49,50,51,52,53,54,55,56,57,58,59,60,61,62] (Figure 2).

Although numerous human microRNAs have been discovered to date, only a few of them were investigated (in terms of measuring their expression level by qPCR) in each study [17]. It brings up the question of how these analysed miRNAs were chosen. The first possibility is an miRNA high-throughput sequencing in a small group of patients (resistant to the therapy compared to controls). It allows one to obtain miRNA expression profiles and select specific miRNAs for further analysis by qPCR [54]. The other commonly applied option is to investigate only miRNAs with a known function, since they are more likely to present an altered expression in the studied group [56,61].

Zhang et al. determined that the circulating miR-223 level was decreased in low-responders to clopidogrel as compared to normal-responders among non-ST-elevation acute coronary syndrome (NTSE-ACS) patients. Moreover, correlation between miR-223 level and platelet reactivity index (PRI) was found [62]. Although Chyrchel et al. described no difference in plasma miR-223 level between NSTE-ACS patients treated with clopidogrel compared to those receiving prasugrel or ticagrelor, pooling all patients’ data confirmed that plasma miR-223 expression significantly correlates with platelet responsiveness, being decreased in low-responders, in line with previous study [50]. Complementary to the above studies, Shi et al. demonstrated that among NSTE-ACS patients miR-223 expression in purified platelets was also downregulated in PRI-determined low-responders. By contrast, miR-96 did not exhibit any correlation with platelet reactivity [58]. Noteworthily, miR-223 plays its role in regulating platelet function through an interaction with 3′ untranslated region of P2Y_12_ mRNA and thereby regulating this ADP receptor expression [63]. Kaudewitz et al. analysed ACS patients (also including STEMI) and found that plasma miR-126 was significantly correlated with platelet reactivity measured by a VerifyNow P2Y_12_ assay. When taking a vasodilator-stimulated phosphoprotein (VASP) assay into consideration, correlation was observed not only in case of miR-126, but also in miR-223 and miR-24 [52]. MiR-126 seems to be a potent regulator of platelet functions with several mechanisms of action [52]. Not only does it modify the expression of P2Y_12_ receptors (like miR-223), but it also downregulates the expression of *ADAM9* in megakaryocytes, a gene encoding protease potentially involved in platelet–collagen interactions [64]. Kok et al. proved that in healthy volunteers under aspirin treatment, intraplatelet miR-19b-1-5p expression was decreased in non-responders characterised by high on-treatment platelet reactivity (HTPR) [53].

Conversely, Li et al. demonstrated that despite platelet miR-21 and miR-126 expression being decreased, as well as miR-150 and miR-223 expression being increased in STEMI patients, compared to healthy individuals, there was no correlation between intraplatelet miRNAs and platelet reactivity—as expressed both by platelet reactivity unit (PRU) and VASP [36]. Using bioinformatics software, Peng et al. anticipated that miR-21, miR-221 and miR-223 could possibly couple with mRNA encoding P2RY12. Taking ACS patients treated with aspirin and clopidogrel into consideration, the expression of the three aforementioned miRNAs in platelets appeared to be decreased in non-responders. Moreover, the receiver operating characteristic (ROC) curves’ analysis showed that platelet miR-21, miR-221 and miR-223 could serve as predictors of clopidogrel antiplatelet therapy responsiveness [57]. As miR-223 had been thoroughly investigated both in circulation and in platelets, another research group decided to examine if the miR-223-3p present in leukocytes was associated with DAPT resistance. Xie et al. proved that there was no significant difference in miR-223-3p levels in leukocytes between non-responders and ultra-responders to clopidogrel therapy [60]. Chen et al. demonstrated that platelet miR-365-3p not only had the highest specificity and sensitivity for detecting HTPR but was also correlated with the SYNTAX score at 24 h and 7 days after DAPT administration among patients with stable angina [49]. Surprisingly, Jäger et al. showed that after the cessation of P2Y_12_ inhibitor therapy in CAD patients, plasma levels of miR-21, miR-126, miR-150 and miR-223 remained unaltered; however, the expression of these miRNAs was affected by the P2Y_12_ inhibitor choice, being increased in patients taking ticagrelor as compared to clopidogrel and prasugrel [51]. Tang et al. evidenced that plasma levels of miR-27a, miR-106a, miR-126, miR-130a and miR-142 were associated with clopidogrel treatment responsiveness being elevated in CAD patients resistant to that therapy (Figure 2). Moreover, miR-142 was highlighted as a potential marker predicting MACE in patients after undergoing PCI [59].

Becker et al. investigated two independent cohorts of NSTE-ACS patients finding that circulating levels of miR-15b-5p, miR-93 and miR-126 were consistently correlated with platelet reactivity [48]. Zhang et al. proved that platelet miR-15b-5p, as well as miR-107, were downregulated in the HPR group of CAD patients compared to the low platelet reactivity (LPR) group of these patients [61]. Liu et al. studied both healthy volunteers and ACS patients, discovering that a decreased level of platelet-derived miR-126 and miR-223 as well as an increased level of miR-150 in individuals with enhanced platelet reactivity is noticeable in each group. In contrast, miR-130 expression also showed a correlation with platelet reactivity similar to miR-126 and miR-223 but only in healthy volunteers [55]. Lin et al. found that platelet miR-411-3p is downregulated, while miR-142-3p and miR-24-3p are upregulated in clopidogrel-resistant CAD patients [54]. Finally, Liu et al. showed that PRI values correlated negatively with platelet miR-223-3p expression among CAD patients [56]. All discussed studies concerning platelet microRNAs have been summarised in Table 1 while research regarding plasma and leukocyte microRNAs has been presented in Table 2.

Different methodologies of sample extraction, microRNA detection and analysis need to be considered while interpreting results of the discussed studies. Firstly, some investigations assessed circulating miRNA whilst the other intraplatelet miRNA. Secondly, platelet reactivity was measured with varied tests, of which the VerifyNow and VASP methods were the most commonly applied. Furthermore, analyses were performed at different timepoints regarding DAPT treatment initiation and administration.

## 6. Future Perspectives

The majority of studies carried out to date enrolled rather small patient groups and judicious interpretation of each finding is indicated. Still, the current evidence could identify meaningful associations which may be useful in guiding target biomarkers’ selection in future larger studies. In particular, the development of microRNA panels, rather than single particle analysis, and identification of high-risk microRNA profiles or ‘fingerprints’ indicative of cardiovascular, as well as impaired pharmacotherapy response hazards appear the most promising targets for future investigations. Nevertheless, standardisation of currently employed analytical methods is warranted before any wider clinical adoption of microRNA as diagnostic tool. The studies addressing the role of microRNA as indices of novel antiplatelet agents’ efficacy should employ the same miRNA for qPCR normalisation, and more rigorous protocols of blood sampling in order to both increase the accuracy of analytical methods and to minimise the impact of time between drug administration and sample collection on measured miRNA levels which could affect the results. Baseline microRNA evaluation before DAPT initiation as well as appropriate longitudinal modelling of serial measurements is strongly recommended [65]. All new findings in this field could contribute to personalized medicine with individualized, rather than a general, therapeutic approach [66,67].

Reliable monitoring of the response to antiplatelet aggregation therapy remains a major challenge. The methods of platelet function assessment developed to date include light transmission platelet aggregometry (LTA), multiple electrode aggregometry, Vasodilator Stimulated Phosphoprotein (VASP) phosphorylation by flow cytometry or VerifyNow system [68]; however, thus far, platelet function testing-oriented randomized clinical trials did not demonstrate clinical benefits of such modified antiplatelet therapy [69]. The potential role of platelet function testing in deescalating antiplatelet therapy and current gaps in evidence were indicated in the 2020 ESC Guidelines [70]. The downsides of the current platelet evaluation modalities drive the search for alternative methods; in this view, there is a promise for miRNAs arrays as a potential novel tools for platelet function assessment and personalized guidance of antiplatelet therapy.

## 7. Conclusions

Despite marked advances in CVD management, there remains a clear need for improved and earlier identification of at-risk subjects to prevent cardiovascular events and safely escalate pharmacotherapy in resistant individuals. Recent evidence suggests correlations between several miRNAs expression and on-treatment platelet reactivity, although there is some heterogeneity between reports, which warrants further studies in the field. There is a need for truly longitudinal biomarker studies with serial blood sampling, paying particular attention to valid methodology of sample preparation, microRNA detection as well as statistical elaboration of the results.

## Figures and Tables

**Table 1:** The summary of recent studies addressing the correlations between platelet miRNAs levels and platelet reactivity.

Authors	Population	Intervention	Comparison	Outcomes	Methods	RNA Used for Normalization
Shi et al. [58]	33 NSTE-ACS pts nondiabetic	300 mg ASA + 300 mg clopidogrel (24 h) or 100 mg ASA + 75 mg clopidogrel (5 days)	group dichotomizedby PRI/PAG	↓ miR-223 in PRI-determined low responders	PRI by VASP phosphorylation and PAG by LTA platelet miRNAs analysis by qPCR	U6
Kok et al. [53]	25 healthy male volunteers 35–65 YO	100 mg ASA OD for 2 weeks + simvastatin 40 mg OD for 6 weeks	blood samples at baseline and after 6 weeks	↓ miR-19b-1-5p expression after aspirin use associated with sustained platelet aggregation	platelet miRNAs analysis by qPCR	previously published normalization panel
Li et al. [36]	40 healthy volunteers and 20 STEMI pts	LD: 600 mg clopidogrel + 300 mg ASA before PCI	healthy volunteers vs STEMI pts correlation between miRNA levels and PR	↓ miR-21, miR-126 ↑ miR-150, m iR-223 in STEMI pts no correlation between miRNAs with PRU or VASP	PRU by VerifyNow + PRI by VASP phosphorylation platelet miRNAs analysis by qPCR	RNU 43
Peng et al. [57]	165 ACS pts	100 mg ASA + 75 mg clopidogrel 300 mg clopidogrel LD to the patients undergoing coronary angiography	21 low-responders and 21 high-responders according to PAG	↑ miR-21, miR-221 and miR-223 in the high-responders compared to the low-responders	relative platelet inhibition based on PAG by LTA at baseline and after 5 days platelet miRNAs analysis by qPCR	U6
Chen et al. [49]	155 stable angina pts and 20 healthy controls	stent implantation + ASA (LD: 300 mg, MD: 100 mg OD) + (clopidogrel (LD: 300 mg, MD: 75 mg OD) or ticagrelor (LD: 180 mg, MD: 90 mg BD) or clopidogrel (LD: 300 mg, MD: 75 mg OD)) + cilostazol (100 mg BD) DAPT for 6 months, then aspirin alone	healthy volunteers subdivision in stable angina pts by PRU	different relationships between miRNAs levels and PRU-determined PR miR-339-3p and miR-365-3p with the highest sensitivity and specificity for detecting HTPR (24 h after drug administration)	PRU by VerifyNow platelet miRNA analysis by qPCR	n.d.
Jäger et al. [51]	62 CAD pts	100 mg ASA OD + (75 mg clopidogrel OD/10 mg prasugrel OD/90 mg ticagrelor BID) cessation of P2Y_12_ inhibitor therapy at baseline	differences in miRNA levels between groups at different time points	cessation of P2Y_12_ inhibitor therapy did not affect platelet miRNA levels differences in miRNA levels between groups (increased in ticagrelor)	MEA by the Multiplate analyser platelet miRNAs analysis by qPCR	cel-miR-39
Liu et al. [55]	430 ACS pts 214 healthy volunteers	100 mg ASA + 75 mg clopidogrel (if PCI, then LD: 300 mg clopidogrel)	10 cases for HPR and 10 for LPR from the healthy group 10 cases for HTPR and 10 for LTPR from ACS pts	↓ miR-223, miR-126 in the pts with HTPR ↑ miR-150 in the pts with HTPR compared to pts with LTPR	PR by TEG platelet miRNAs analysis by qPCR	U6
Zhang et al. [61]	50 CAD pts after PCI	Before PCI: 75 mg clopidogrel for 6 days or LD 300 mg clopidogrel and 300 mg ASA After PCI: 100 mg ASA OD + 75 mg clopidogrel OD	28 pts in the HPR group and 22 pts in the LPR group	↓ miR-107, miR-15b-5p it the pts with HPR	PRU by VerifyNow platelet miRNAs analysis by qPCR	U6
Lin et al. [54]	60 CAD pts after PCI	100 mg ASA OD + 75 mg clopidogrel OD	30 pts in clopidogrel resistance group (CR) and 30 pts in control group	↓ miR-411-3p in the CR group ↑ miR-142-3p, miR-24-3p in the CR group	IPA by VerifyNow platelet miRNA analysis by qPCR	n.d.
Liu et al. [56]	37 CAD pts after PCI	100 mg ASA + clopidogrel (LD: 300 mg, MD: 75 mg OD)	correlation between PRI values and miRNA expression levels	PRI values negatively correlated with miR-223-3p expression	PRI by VASP phosphorylation flow cytometry platelet miRNA analysis by qPCR	U6

↑—increased, ↓—decreased.

**Table 2:** The summary of recent studies addressing the correlations between plasma and leukocyte miRNAs levels and platelet reactivity.

Authors	Population	Intervention	Comparison	Outcomes	Methods	RNA Used for Normalization
Zhang et al. [62]	62 NSTE-ACS pts with troponin negative	300 mg ASA + 300 mg clopidogrel (24 h) or 100 mg ASA + 75 mg clopidogrel (5 days)	group divided into subgroups compared with each other	↓ plasma miR-223 in PRI low-responders miR-223 expression correlated with PRI	PRI by VASP phosphorylation flow cytometry plasma miRNAs analysis by qPCR	human-5 s-rRNA
Chyrchel et al. [50]	21 NSTE-ACS men with an uncomplicated hospital course	(75 mg clopidogrel OD or 10 mg prasugrel OD or 90 mg ticagrelor BID) + 75–100 mg ASA OD	correlation between plasma miR-223 and PAG PAG compared between clopidogrel & prasugrel/ticagrelor	↓ PR in pts on prasugrel or ticagrelor compared to clopidogrel ↑ miR-223 with increased DAPT responsiveness	PR measured by MEA plasma miRNAs analysis by qPCR prasugrel and ticagrelor combined in miRNAs analysis	miR-16
Kaudewitz et al. [52]	125 ACS pts 30 days after onset of ACS	ASA + clopidogrel/prasugrel/ticagrelor	correlations between miRNA levels and platelet functions	no correlation between miRNAs and PAG miR-24, miR-126, miR-191, miR-223 correlated with PRU miR-126 correlated with PRI	PRU by VerifyNow and PRI by VASP phosphorylation and PAG by LTA plasma miRNAs analysis by qPCR	cel-miR-39*
Xie et al. [60]	188 NSTE-ACS pts after elective PCI	100 mg ASA + 75 mg clopidogrel	47 ultra-responders (UR) and 47 non-responders (NR) according to PAG (only 24 and 36 samples analysed, respectively)	no difference in hsa-miR-223- 3p between the NR and UR no significant correlation between miR-223-3p PAG	PAG as a value of electronic impedance (Ω) between the two electrodes immersed in a diluted sample leukocyte miR-223-3p analysis by qPCR	U6
Tang et al. [59]	115 CAD pts + 1199 CAD pts as a prospective cohort	100 mg ASA + 300 mg clopidogrel LD	pts divided into 4 subgroups according to ARU and PRU	↑ miR-142 associated with a high risk of MACE ↑ miR-126, miR-142, miR-130a, miR-27a miR-106a in pts resistant to clopidogrel therapy	PRU and ARU by VerifyNow plasma miRNAs analysis by qPCR plasma samples mixed from pts of a given subgroup	cel-miR-39
Becker et al. [48]	878 NSTE-ACS pts + 96 pts NSTE-ACS in corroborative cohort	100 mg ASA + (75 mg clopidogrel/10 mg prasugrel)	nested case-control study correlation between PR and miRNA levels	miR-15b-5p, miR-93 and miR-126 associated with PR	PRU by VerifyNow or PRI by VASP phosphorylation or Multiplate ADP test assayplasma miRNAs analysis by qPCR and non-targeted miR-Seq analysis	3 most stable endogenous miRNAs

↑—increased, ↓—decreased, ACS—Acute Coronary Syndrome, ARU—aspirin reaction unit, ASA—acetylsalicylic acid, BID—twice daily, CAD—Coronary Artery Disease, DAPT—dual antiplatelet therapy, HPR—high platelet reactivity, HTPR—high on-treatment platelet reactivity, IPA—inhibition of platelet aggregation, LD—loading dose, LPR—low platelet reactivity, LTA—light transmittance aggregometry, LTPR—low on-treatment platelet reactivity, MACE—major adverse cardiovascular events, MD—maintenance dose, MEA—multiple electrode aggregometry, MEA—Multiple electrode aggregometry, miR/miRNA—microRNA, n.d.—no data, NSTE-ACS—non-ST-Elevation Acute Coronary Syndrome, OD—once daily, PAG—ADP-induced platelet aggregation, PCI—percutaneous coronary interventions, PR—platelet reactivity, PRI—platelet reactivity index, PRU—platelet reactivity unit, pts—patients, qPCR—quantitative Polymerase Chain Reaction, STEMI—ST-Elevation Myocardial Infarction, TEG—thromboelastogram, VASP—vasodilator-stimulated phosphoprotein, YO—years old.
